# Lithium-associated transcriptional regulation of *CRMP1* in patient-derived olfactory neurons and symptom changes in bipolar disorder

**DOI:** 10.1038/s41398-018-0126-6

**Published:** 2018-04-18

**Authors:** Charlee K. McLean, Soumya Narayan, Sandra Y. Lin, Narayan Rai, Youjin Chung, MariaMananita S. Hipolito, Nicola G. Cascella, John I Nurnberger, Koko Ishizuka, Akira S. Sawa, Evaristus A. Nwulia

**Affiliations:** 1 Department of Psychiatry and Behavioral Sciences, Howard University, Washington, DC USA; 2 Department of Psychiatry, Johns Hopkins University, Baltimore, MD USA; 3 Department of Otolaryngology-Head and Neck Surgery, Johns Hopkins University, Baltimore, MD USA; 4 Department of Psychiatry, Sheppard Pratt Health Systems, Baltimore, MD USA; 5 Department of Psychiatry, Indiana University, Bloomington, IN USA

## Abstract

There is growing evidence that lithium used in the treatment of bipolar disorder (BD) affects molecular targets that are involved in neuronal growth, survival, and maturation, but it remains unclear if neuronal alterations in any of these molecules predict specific symptom changes in BD patients undergoing lithium monotherapy. The goals of this study were to (a) determine which molecular changes in the olfactory neurons of symptomatic patients receiving lithium are associated with antimanic or antidepressant response, and (b) uncover novel intraneuronal regulatory mechanisms of lithium therapy. Twenty-two treatment-naïve non-smoking patients, with symptomatic BD underwent nasal biopsies for collection of olfactory tissues, prior to their treatment and following a 6-week course of lithium monotherapy. Sixteen healthy controls were also biopsied. Combining laser capture microdissection with real-time polymerase chain reaction, we investigated baseline and treatment-associated transcriptional changes in candidate molecular targets of lithium action in the olfactory neuroepithelium. Baseline mRNA levels of glycogen synthase kinase 3 beta (*GSK3β*) and collapsin response mediator protein 1 (*CRMP1*) genes were significantly associated with BD status and with severity of mood symptoms. Among BD subjects, treatment-associated downregulation of *CRMP1* expression was most predictive of decreases in both manic and depressive symptoms. This study provides a novel insight into the relevance of *CRMP1*, a key molecule in semaphorin-3A signaling during neurodevelopment, in the molecular mechanism of action of lithium, and in the pathophysiology of BD. It supports the use of human-derived olfactory neuronal tissues in the evaluation of treatment response of psychiatric disorders.

## Introduction

Development of new treatments for patients with bipolar disorder (BD) is often stalled by limited knowledge of the neuronal molecular mechanisms of mood stabilizing medications in living patients. Lithium has been shown in preclinical studies to alter the activity of a large number of genes involved in neuronal growth, differentiation, and survival^1–8^. However, it remains largely unknown if lithium-induced alterations in any of these molecules within the nervous system are actually associated with clinical response in humans^7^. Moreover, while direct and indirect post-translational effects of lithium on these candidate enzymes have been documented by several groups, its effects over their transcriptional regulation have not yet been fully addressed, particularly in humans. Such inquiry would uncover new molecular mechanisms of lithium that could potentially lead to the development of clinically relevant novel drugs that may have fewer side effects than lithium, which affects a broader number of targets.

Elucidating the mechanisms of mood-stabilizing medications would ideally require investigating treatment-induced molecular changes in neuronal tissues of living patients, but it is impractical to obtain repeated brain biopsies of BD patients while undergoing therapy. Postmortem brain tissue has been useful to our understanding of biomarkers of BD, but they cannot provide prospective information on dynamic changes in emotions, cognition, and drive. Lymphocytes and other blood cells obtained from living patients may be useful, but transcriptome is poorly correlated between the brain and blood^9^. The focus of our study is to directly detect a neuronal molecular signature related to lithium response in patients with BD by developing a paradigm that allows for feasible repetitive live sampling of nervous tissue while patients undergo lithium therapy.

The olfactory neuroepithelium has received increased interest as a window to brain mechanisms in complex psychiatric diseases^10–14^. Furthermore, olfactory neuropithelium contains olfactory receptor neurons at different stages of development, and provides a unique resource for investigation of state and trait intraneuronal molecular markers of neuropsychiatric diseases^15^. One significant limitation to the use of olfactory tissue is the substantial contamination of nasal biopsies with non-neuronal cells. This challenge has been overcome through an innovative approach to obtain enriched neuronal cell populations by combining nasal biopsies with laser-capture microdissection (LCM)^16^. By further incorporating quantitative real-time polymerase chain reaction (RT-PCR) with this latter approach, we recently developed a novel platform for investigating intraneuronal molecular signatures in olfactory neurons^17^.

Glycogen synthase kinase 3β (GSK3β), a well-documented candidate gene of BD is abundantly expressed in the olfactory bulb (OB), where it regulates the axonal stability in olfactory neurons^18,19^. While evidence for relevance of GSK3β in BD has historically focused on its activity levels, recent studies in human brain autopsies suggest that its mRNA levels are also altered in the prefrontal cortex of patients with BD, compared to non-bipolar controls^20^. Moreover, recent evidence that lithium modulates *GSK3β* transcription in vitro and in vivo in animal models offers a new and complementary explanation for GSK3*β* modulation, but also raises a novel question whether such transcriptional regulation in the neurons of BD patients actively taking lithium predicts clinical outcome. Lithium-induced inhibition of *GSK3β* has also been found to mediate mRNA levels of AKT1 in a dose-dependent manner^21^, providing another complementary explanation for AKT1 modulation in BD. In the olfactory system, AKT1 modulates neurite outgrowth during development^22^. Protein kinase C, especially PKC*ε* is richly expressed in olfactory epithelium, where it sensitizes the olfactory adenylate kinase and contributes to neuronal excitability^23^. A recent study on induced pluripotent stem cells (iPSCs)-derived neurons of BD patients and controls revealed elevated mRNA levels of PKC genes in concert with differential hyperexcitability in BD neurons^24^, further cementing the potential relevance of transcriptional regulation of PKC to BD.

In this current study, we used the olfactory neuroepithelium platform to conduct transcriptional profiling of *AKT1*, *PKCε*, and *GSK3β* in BD patients at baseline (i.e., before initiation of lithium therapy) and after a 6-week course of lithium therapy. Based on preclinical evidence that lithium activates *AKT1*^5^ and inhibits *GSK3β* and *PKCε*^8^, we hypothesized that: (1) BD status and symptom severity at baseline would be directly associated with baseline mRNA levels of *GSK3β* and *PKCε*, but inversely related to baseline mRNA levels of *AKT1*; (2) lithium treatment would result in downregulation of mRNA levels of *GSK3β* and *PKCε*, and upregulation of mRNA levels of *AKT1*; and (3) lithium-associated transcriptional changes of these genes would be significantly associated with reduction in symptoms.

As we were also interested in identifying novel molecules that are involved in efficacy of lithium treatment, we explored the expression of collapsin response mediator protein 1 (CRMP1), a GSK3β substrate in BD. The *CRMP1* gene was recently reported to be differentially expressed in patients with schizophrenia and BD compared to controls^16^, and CRMPs are differentially expressed in neuronal cells that undergo neurogenesis life-long, such as the olfactory epithelium and the dentate gyrus of the hippocampus^25^. In addition, we reported that CRMP1 was associated with odor identification, physical and social anhedonia in patients with schizophrenia^26^. Guided by these pieces of evidence, we also explored whether CRMP1 expression is a putative marker of clinical response from lithium.

## Patients and methods

### Study population

Adult patients with BD (*N* = 30) and healthy controls (*N* = 20) (18–65 years old) were recruited from inpatient wards, outpatient mental health clinics, and private provider clinics at Howard University, as well as from surrounding community clinics and populations in the Washington, DC, metropolitan region. The population in this catchment area is 94% of African–American origin. A DSM-IV diagnostic interview was conducted on all subjects by the study psychiatrist (Dr. Evaristus A. Nwulia, one of co-authors of this paper), using the Diagnostic Interview for Genetic Studies, Version 4 (DIGS 4.0), which we previously used for the diagnosis of BD in genome-wide genetic studies of BD in both Europeans and African Americans^27^. BD subjects were defined as those that met DSM-IV diagnostic criteria for lifetime bipolar I (BDI) or bipolar II disorder (BDII), based on the DIGS; while control subjects are those who did not meet criteria for BDI, BDII, or BD, not otherwise specified (BD-NOS). Given the primary objective of this study to investigate molecular markers of clinical response (or reduction of symptom severity) in BD, we only included BD subjects who were persistently symptomatic for depression, hypomania, or mania for two or more weeks prior to baseline studies. Screening for depressive and manic/hypomanic symptom severity was conducted by trained clinical research staff (Dr. Maria Hipolito, one of co-authors of this paper) using the Montgomery-Asberg Depression Rating Scale (MADRS)^28^ and the Young Mania Rating Scale (YMRS)^29^. The MADRS is a well-validated clinician-administered 10-item measure of depression severity, with total scores ranging from 0 to 60; higher scores connoting greater severity^28^. The YMRS is a well-validated 11-item questionnaire used by clinicians to measure the severity of mania in adult subjects with BD; and the scores also range from 0 to 60. Inclusion criteria for symptomatic BD subjects included a score of ≥10 out of 60 on MADRS or a score of ≥10 out of 56 on YMRS, and raters were blind to the diagnosis of each study participant. Inter-rater reliability for both scales was >0.96. Exclusion criteria for all subjects included a history of current tobacco smoking or current substance use disorder, diagnosis of antisocial personality disorder, other major Axis 1 disorders (except for history of childhood separation anxiety), mental retardation, seizure and other neurological disorders, renal diseases, cardiac arrhythmias, hypothyroidism, or pregnancy in females. Study procedures were approved by the Howard University and Johns Hopkins University Institutional Review Boards (IRB) for the Protection of Human Subjects for the ethical conduct of research involving human subjects in accordance with the Declaration of Helsinki. All participants provided informed consent to participate following a complete discussion of the study. All subjects received thyroid function and renal function tests during screening, while urine drug tests were obtained at baseline and on the 6th week. Women, in addition, received pregnancy tests at baseline and on the 6th week of the study. All participants provided informed consent according to protocols approved by the IRB of Howard University and Johns Hopkins University. Out of 30 BD subjects initially enrolled for this study, 22 were treatment-naïve at the time of inclusion and eight were recently medicated (see Supplementary Figure 1 for study flowchart). Three of the latter joined the study after successfully completing a 21-day wash-out period during which they received cognitive–behavioral therapy and daily psychiatric evaluation. Two controls were excluded for testing positive for illicit drugs on repeat drug screen. Altogether, 25 BD subjects and 18 controls were eligible for baseline nasal biopsy (Supplementary Figure 1). All subjects included in this analysis are of African–American origin.

### Lithium monotherapy

Due to ethical concerns raised by the IRB, including the risk of self-harm, all symptomatic BD subjects were required to receive treatment, so that the molecular effects of the treatment were determined by comparing pre- and post-treatment molecular changes within-individuals. Lithium carbonate was orally administered to all 22 BD subjects, starting at a dose of 300 mg on the first day, reaching 900 mg on the first week, and titrated upward (if necessary) to attain a serum lithium level in the range of 0.6–1.2 mEq/l by the second week. Subjects who did not adhere or did not tolerate the required dose adjustments (*N* = 3) were included only for baseline studies (Supplementary Figure 1). Serum lithium levels were obtained twice a week in the first 2 weeks to guide dose adjustment. This was to ensure that desired serum levels (i.e., 0.6–1.2 mEq/l) were attained for patients by the end of the second week. Subsequently, serum levels were checked in the 4th and 6th weeks. Patients were seen weekly at the Howard University Mood Disorders Clinic for review of laboratory results, side effects, and for dose adjustment.

### Nasal biopsy

Nasal biopsies were performed at the Johns Hopkins Otolaryngology Clinic on all volunteers on the first day of the study (prior to the first dose of lithium for BD cases), and 6 weeks later. The procedure was previously described in detail by Kano et al.^30^. Three 1-mm tissue blocks were removed from each nostril. After the biopsy, the subjects were observed in the clinic for 15–30 min before transporting the subjects back to District of Columbia (DC).

### Molecular methods

Methods for the preparation of tissue blocks, cryosectioning, LCM, isolation of total RNA, RNA quality control, complimentary DNA (cDNA) synthesis, and RT-PCR have been described in detail in our previous publication^17^. The samples have no information on the case-control status of the participants who provided the samples, so that technicians performing all molecular studies were blinded to this information. We provide summarized descriptions below.

### Preparation of tissue blocks, cryosectioning, and LCM

Immediately prior to biopsy, standard sized cryomolds were filled with tissue-freezing medium (Tissue-Tek O.C.T Compound). Autoclaved tweezers were used to mount fresh tissue into the freezing medium; the tissue was frozen on dry ice, and blocks were subsequently stored at −80 °C. Polyethylene naphthalate (PEN) membrane glass slides were prepared immediately prior to cryosectioning. Slides were sprayed with RNase Zap solution for 5 min, then rinsed in diethylpyrocarbonate (DEPC) water and air-dried for 30 min. Dried slides were activated under UV light for 30 min, and each block was cryosectioned entirely at 30 µm. Slides were kept on dry ice, and sections were kept frozen as much as possible. Prior to LCM, slides were immediately dehydrated: frozen slides were left to thaw for 10 s at room temperature. Photoactivated localization microscopy (PALM) microscope was turned on and the parameters (i.e., magnification, energy, and focus) were adjusted as necessary to obtain optimal results in identifying the olfactory neuronal layer. On the microscope field, the thin surface olfactory neuron layer, which has a dense columnar appearance, can be distinguished from underlying sub-mucosa without the use of staining procedure (Supplementary Figure 2). Upon completion of dissection, sample lysates were immediately subjected to vortex, kept on dry ice and stored at −80 °C for total RNA isolation.

### Isolation of total RNA, RNA quality control, and cDNA synthesis

We have previously established and published the benchmark for RNA Integrity Number (RIN) for reproducible mRNA studies in this platform^16,17^ (https://www.jove.com/video/51853/olfactory-neurons-obtained-through-nasal-biopsy-combined-with-laser). Briefly, total RNA isolation was carried out using RNAqueous Micro Kit with DNase-I inactivation by following the manufacturer’s protocol. We then measured the RNA concentration and established RNA quality with RIN using Bioanalyzer. Note, there was no statistical correlation between RIN values and mRNA levels (Supplementary Figure 3). cDNA was synthesized using the Invitrogen SuperScript III First Strand Synthesis Kit. The annealing step was performed by combining 0.1–1.0 ng of RNA and RNase free water to a total volume of 8 μl per tube. Master mix solutions were prepared according to manufacturer's specifications. Subsequently, 9.5 µl master mix and 0.5 µl of commercially prepared Reverse Transcriptase (RT) were added to each tube, while 0.5 µl RNase-free water was added to control tubes instead of RT. The RT reaction was run with the first incubation at 50 °C for 50 min, then 85 °C for 5 min, and a final 4 °C hold, with no cycling. Samples were diluted by 5× with water and aliquoted, for storage at −80 °C until PCR.

### RT-PCR for confirmation of neuronal enrichment and for candidate genes

*GAPDH* was used as an internal control. We found no differences between BD and controls in the expression of this housekeeping gene (Supplementary Figure 4). The *GAPDH* primer sequence for amplification was forward, 5′-CTGTCGGACCTGGCCAAG-3′; reverse, 5′-CACCCTCGCCAAAGGTGA-3′. To further confirm neuronal enrichment in our laser-captured samples, we compared the olfactory marker protein (*OMP*) expression in microdissected tissue to *OMP* expression in undissected tissue. The following *OMP* primer sequence was used: forward, 5′-CTGTCGGACCTGGCCAAG-3′; reverse, 5′-CACCCTCGCCAAAGGTGA-3′. A PCR plate was prepared by adding 7 µl of master mix and 3 µl of cDNA in each well. Each plate was centrifuged for 5 min at 300×*g* (1300 rpm). RT-PCR reactions were run using the default thermal cycling conditions specified for commercial systems, such as SYBR GreenER qPCR. PCR reaction data for *OMP* were analyzed for microdissected and undissected tissue to select samples that showed ≥2-fold neuronal (i.e., *OMP*) enrichment, to be used for the study of the candidate genes of interest^17^.

Gene expression was analyzed with the following Taqman assays: *GSK3β* (assay ID: Hs01047719_m1), *AKT1* (assay ID: Hs00178289_m1), *PKCε* (assay ID: Hs00178455_m1), *CRMP1* (assay ID: Hs00609716_m1), and *GAPDH* (assay ID: Hs02758991_g1). Master mix preparations were made for each probe in individual tubes according to established specifications^17^. Probes of interest were FAM-labeled, and GAPDH was VIC-labeled. PCR plates were prepared with 8 µl master mix and 2 µl of cDNA per well. Plates were centrifuged for 5 min at 300×*g* (1300 rpm). An RT-PCR reaction was run using the default thermal cycling conditions and following the gene expression master mix protocol provided by the manufacturer.

### Statistical analysis

Descriptive statistics, including tests of normality and distribution of variables, visualization of summary statistics, and all regression analyses were conducted in Stata 14^31^. In terms of baseline studies comparing mRNA levels in BD vs. controls, four comparison tests (i.e., four genes) are being conducted. But we consider these tests as exploratory, since the primary goal of the study is to determine the linear effects of lithium-associated gene changes on affective changes; as such, we do not apply multiple comparison tests to baseline studies. Student's *t*-test was used to compute mean estimates of continuous variables between groups (e.g., BD vs. controls) at baseline; *P* values for group differences in non-normal continuous variables were derived from a Mann–Whitney rank test; *χ*^2^ and Exact tests were used to compare categorical variables (e.g., sex). In addition to comparing BD vs. controls on baseline mRNA levels, linear relationships between baseline dimensional mood severity ratings (i.e., YMRS and MADRS) and baseline mRNA levels were explored through linear regression models, with robust variance–covariance approach, which is robust to both differences in variances between groups and to violation of normality assumption. However, YMRS and MADRS scores of controls needed to be combined with those of cases in the latter regression analyses due to extreme deviation of YMRS and MADRS scores—within each case-control group—from normality. This approach ensured more reliable estimation of standard errors for regression coefficients and minimized the threat of type II errors from sample size of the BD group. Linear mixed (fixed-random) effect models were used to estimate the effect of lithium treatment on repeat outcome measures, such as pre- and post-treatment mRNA levels and pre- and post-treatment depression (MADRS) and mania (YMRS) ratings. In terms of linear effects of treatment on mRNA levels, statistical analysis was conducted only for genes that showed significant baseline difference between BD subjects and controls. To avoid multiple comparisons, the linear model is based on pre-specified hypothesis that the effect of treatment on the genes is conditional age, sex, and any other confounders (from baseline summary statistics analysis). Such that, if only two genes differentiate BD from controls at baseline, then only two pre-specified (hypothesis-driven) comparison tests are conducted for treatment effects. To derive statistical inferences for the mixed effect models, we used denominator-degree of freedom (DDF) adjustment for small sample sizes. For all tests, statistical significance was accepted when *P* ≤ 0.05. From a preceding pilot study, mRNA levels of *GSK3β* measured in three cases and three controls were used to generate a statistical rationale for the sample size goals of this study (Supplementary Figure 5). A total of 16 participants were sufficient to detect true differences in *GSK3β* mRNA levels between cases and controls, with 0.80 power and false-positive rate of 0.05.

## Results

### Baseline demographic and clinical variables of study sample

Table 1 highlights the clinical and demographic features of eligible BD patients (*N* = 22) and controls (*N* = 16) who had complete molecular and clinical data at the baseline. The control group had disproportionately more females and attained higher educational levels. Group differences in age did not meet the threshold for statistical significance due to wide distributions in age, highlighted by the large standard deviations. Out of the 22 BD subjects, 19 were BDI, based on a lifetime episode of at least one manic episode; and three subjects were BDII, based on lifetime history of hypomania and recurrent depressive episodes. Mean (SD) duration of current episode is 39.3 (10.6) days. Among BD subjects, severity ratings in YMRS range from 0 (i.e., those with just depressive symptoms) to 35; while MADRS severity ranged from 1 (i.e., those with purely manic/hypomanic symptoms) to 33. The mean and standard deviations for YMRS and MADRS are depicted in Table 1. The proportion of BD subjects meeting the inclusion criteria for mania/hypomania, depressed, and mixed (mania and depression) are also depicted in Table 1.

**Table 1:** Demographic and clinical features of study participants at baseline

Measures	Bipolar disorder (*N* = 22)		Control (*N* = 16)	
	Mean	SD	Mean	SD
Age (years)	33.64	13.60	41.62	12.25
Education (years)**	13.00	2.11	16.0	2.90
MADRS***^a^	16.82	9.07	1.19	2.51
YMRS***^b^	17.54	7.23	0.62	1.36
Episode duration (weeks)	20.41	20.02		
	*N*	%	*N*	%
Female*	18	81.82	8	50.00
Manic/hypomanic	19	86.36		
Depressed	15	68.18		
Mixed	12	54.54		
Smoking (past)	5	23.64	4	22.50
Psychotropic (past)***	9	39.09	0	0.00

*SD* standard deviation, % percent proportion

* *P* < 0.05; **P < 0.01; ***P < 0.001

^a^MADRS range = 2–35; median (interquartile range) = 18 (10–22)

^b^YMRS range = 5–29; median (interquartile range) = 18.5 (15–23)

### Association among baseline gene expression, BD, and mood severity

**Fig. 1:**
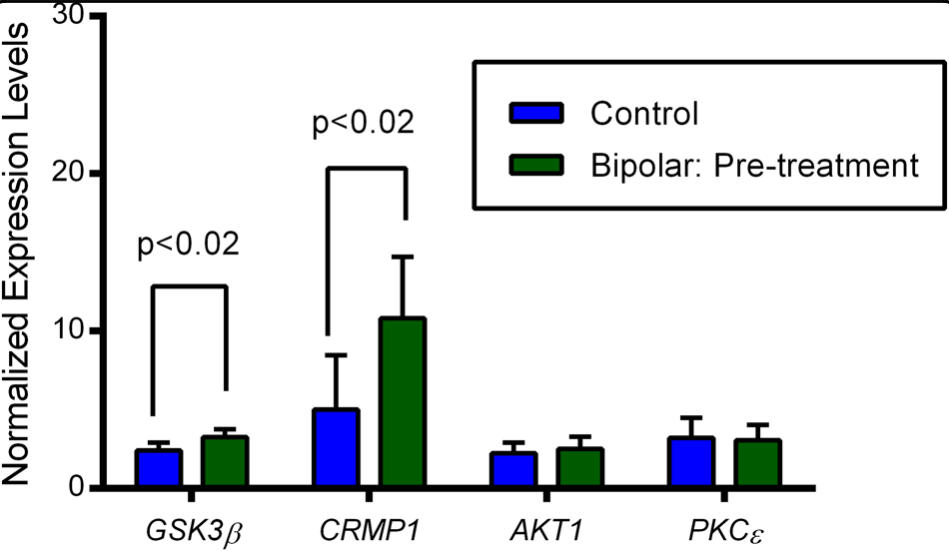
Baseline Normalized Expression Levels of GSK3β, CRMP1, AKT1 and PKCein Patients with Bipolar Disorder Compared to Controls. Normalized expression levels of GSK3β, CRMP1, AKT1 and PKCein bipolar subjects compared to controls. GAPDH was used for an internal control for normalization of expression levels of these candidate genes. Significant differences (*P* < 0.02) were observed in the expression levels of GSK3β and CRMP1 in symptomatic bipolar subjects compared to controls

Results of the comparison of BD cases to controls on baseline mRNA levels for *GSK3β*, *CRMP1*, *AKT1*, and *PKCε* are highlighted in Fig. 1. As shown, baseline mRNA levels of *GSK3β* and *CRMP1* were significantly higher in BD versus controls. No significant associations were observed for *AKT1* or *PKCε* expression. Results of simple and multivariate regression analyses examining the associations between gene transcriptional levels and YMRS and MADRS scores are displayed in Table 2. Multivariate analysis adjusting for sex, age, and education (which were disproportionately distributed in cases vs. controls) revealed a stronger association for *CRMP1* for YMRS (*P* < 0.009) and a trend in association for MADRS (*P* < 0.09). In contrast, multivariate analysis did not change the estimates for *GSK3β*. No associations were observed between mood symptoms and either *AKT1* or *PKCε* (Table 2).

**Table 2:** Association between baseline *CRMP1*, *GSK3β*, *AKT1*, and *PKCε* mRNA levels and severity of mood symptoms

	YMRS			MADRS		
	*β*	95% CI	*P*	*β*	95% CI	*P*
*GSK3*β (*N* = 38)						
Unadjusted^a^	2.84	0.36–5.27	<0.02	0.89	−2.35–4.14	<0.60
Adjusted^a,b^	2.81	0.60–5.51	<0.02	0.76	−2.46–3.99	<0.70
*CRMP1* (*N* = 35)						
Unadjusted^a^	0.45	0.11–0.79	<0.02	0.34	−0.10–0.79	<0.20
Adjusted^a,b^	0.48	0.13–0.84	<0.009	0.38	−0.05–0.80	<0.09
*AKT1* (*N* = 37)						
Unadjusted	1.24	−0.76–3.24	<0.30	1.15	−1.76–4.06	<0.30
Adjusted^b^	1.39	−0.69–3.47	<0.20	1.35	−1.66–4.35	<0.40
*PKCε* (*N* = 36)						
Unadjusted	−0.38	−1.93–1.17	<0.70	−1.1	−2.42–0.22	<0.10
Adjusted^b^	−0.25	−1.72–1.23	<0.80	−0.94	−2.34–0.46	<0.20

*CI* confidence interval

^a^Significant association between the baseline normalized expression levels and severity of mood symptoms

^b^Adjusted for differences in age and sex, and education for all subjects for the different targeted genes

### Effect of 6 weeks of lithium therapy on *GSK3β* and *CRMP1* mRNA levels

**Fig. 2:**
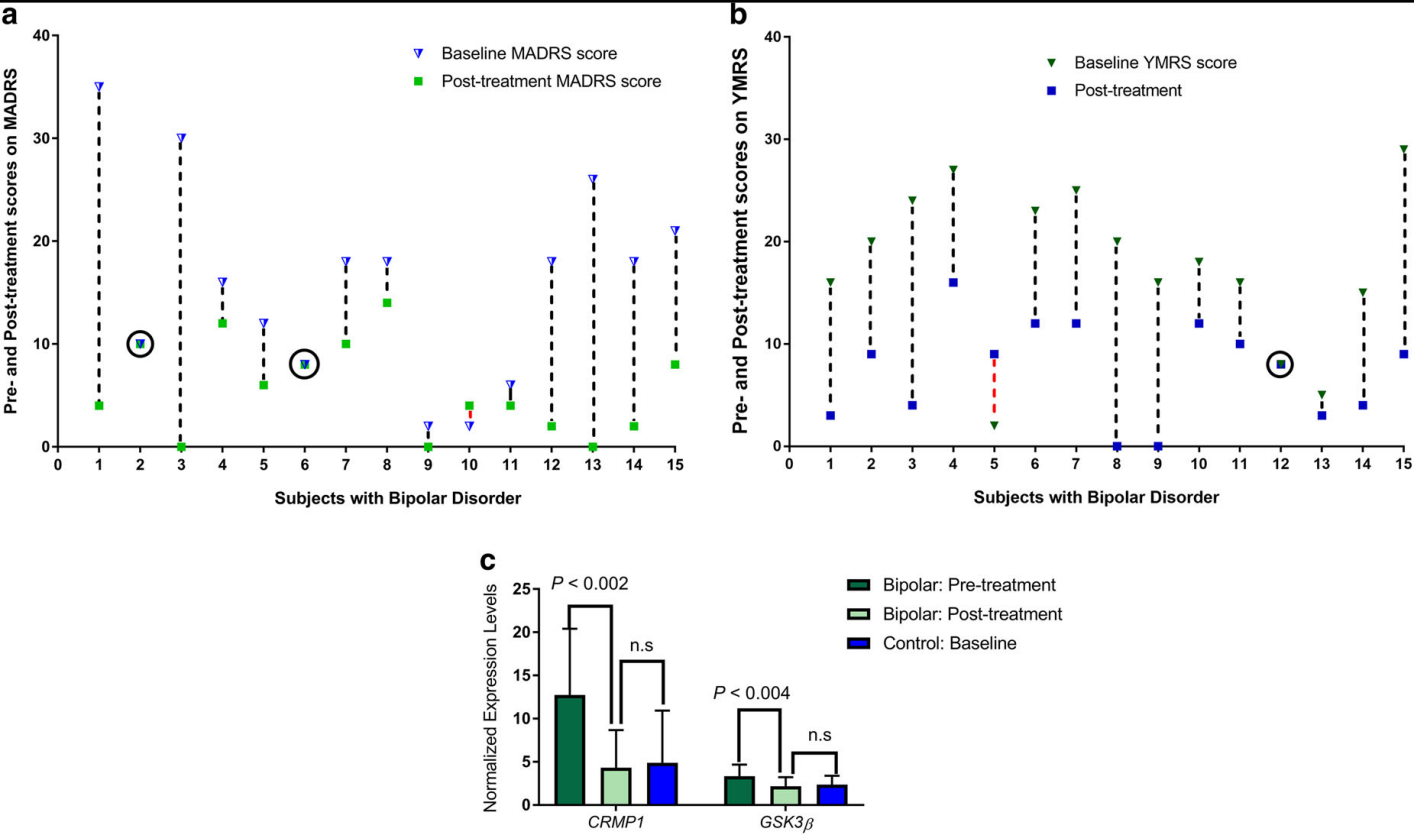
Lithium-Associated Changes in Affective Symptoms and in Gene Transcriptions. **a** Individual-level changes in the severity of depression based on scores on the MADRS scale obtained on each BD subject at baseline and after 6 weeks of lithium therapy. Baseline MADRS scores are represented in Blue triangles, post-treatment scores are represented in Green squares, and the dash lines between scores represent the differences between pre- and post-treatment depression severity. Apart from onesubject (the Red dash), all subjects experienced improved symptoms. **b** Individual-level changes in severity of mania/hypomania based on scores on the YMRS scale obtained on each BD subject at baseline and after 6 weeks of lithium therapy. Baseline YMRS scores are represented in Green triangles, post-treatment scores are represented in Blue squares, and the dash lines between scores represent the differences between pre- and post-treatment depression severity. Red dash indicates worsening severity. Note: both (2a and 2b) illustrate considerable improvements in mood symptoms, but also highlight ceiling effects for people with low pre-treatment levels in MADRS or YMRS scores. The degree of change is dependent on the starting (i.e. baseline) scores, which necessitates use of repeat-measures approach to statistical analysis of treatment effects. **c** Pre- and Post-treatment normalized mRNA levels of CRMP1 and GSK3β among BD subjects and controls who received 6 weeks of lithium therapy. Among BD subjects, mRNA levels were significantly downregulated post-treatment. Pre- and post-treatment differences in transcriptions were not observed for controls

All BD patients achieved plasma lithium levels ≥0.6 mEq/l. The mean lithium level (interquartile range) in the entire group is 0.87 (0.64–0.92) mEq/l. Individual-level changes in the severity of depression and mania/hypomania are depicted in Fig. 2a, b, respectively. These figures illustrate considerable improvements in mood symptoms, but also highlight ceiling effects for people with low pre-treatment levels in MADRS or YMRS scores. Analysis of changes in mRNA levels within individuals and comparison of pre-treatment to post-treatment levels among cases shows that lithium therapy results in consistent downregulation of *GSK3β* and *CRMP1* transcriptions. As shown in Fig. 2c, lithium therapy led to marked reductions in mRNA levels of *CRMP1* and *GSK3β*, *P* < 0.002 and *P* < 0.004, respectively. The linear associations between pre- and post-treatment lithium levels and within-individual (pre- and post-treatment) changes in mRNA levels of *GSK3β* and *CRMP1* are depicted in Supplementary Table 1: a unit increase in plasma lithium is associated with 0.69 (95% CI, 0.11–1.28) unit decrease in *GSK3β* levels; and a unit increase in plasma lithium is associated with 5.89 (95% CI, 0.18–11.61) unit decrease in *CRMP1* levels. Comparison of mean post-treatment mRNA levels of *GSK3β* and *CRMP1* among BD cases to the mean baseline expression results for corresponding genes among healthy controls suggests that lithium therapy probably results in normalization of expression of these two molecules, i.e., reduction of mRNA levels to the range of healthy controls (Fig. 2c). In contrast to BD cases, comparisons of baseline and 6th-week mRNA levels of GSK3β and CRMP1 among six control subjects, who repeated biopsy, did not reveal this pattern of reduction of the second (i.e., 6^th^ week) mRNA levels from their baseline levels (Supplementary Figure 6).

### Effect of lithium-associated molecular changes on clinical symptoms

To determine the effect of lithium-associated changes in mRNA levels of *GSK3β* and *CRMP1* on clinical response, we modeled changes in YMRS and MADRS scores as a function of change in mRNA levels of each gene, comparing 6th week to baseline levels. Although the confounding variables (age, sex, and education) are fixed within individuals, we controlled for them to be consistent with the multivariate model of baseline analysis. As shown in Table 3, one unit decrease in mRNA levels of *GSK3β* was associated with 2.19 (*P* < 0.05) and 2.43 (*P* < 0.02) point decreases in YMRS for the unadjusted and adjusted models, respectively. The effect of lithium-associated downregulation of *GSK3β* onthe reduction of MADRS scores was not statistically significant. On the other hand, in unadjusted models, a unit decrease in the mRNA levels of *CRMP1* was associated with significant decreases for both YMRS (*P* < 0.02) and MADRS (*P* < 0.04) scores. Adjusting for gender, age, and education did not alter the direction of these effects or their strength of association. The strength of statistical associations between *CRMP1* and mood severity symptoms did not diminish when we repeated the linear mixed effect analysis that excluded *CRMP1* data from two subjects with undetectable post-treatment mRNA CRMP1 levels (Table 3).

**Table 3:** Effect of lithium-associated downregulation of *CRMP1* and *GSK3β* and changes in mood severity following 6 weeks of treatment

	YMRS			MADRS		
	*β*	95% CI	*P*	*β*	95% CI	*P*
*GSK3β *(*N* = 15)						
Unadjusted	2.19	−0.09–4.28	<0.05	−0.61	−3.11–1.88	<0.70
Adjusted^a^	2.43	−0.44–4.41	<0.02	−0.35	−2.92–2.21	<0.80
*CRMP1 *(*N* = 13)						
Unadjusted	0.37	0.06–0.68	<0.02	0.42	0.03–0.80	<0.04
Adjusted^a^	0.36	0.06–0.67	<0.02	0.46	0.08–0.84	<0.02
Unadjusted^b^	0.54	0.15–0.94	<0.007	0.47	−0.06–1.00	<0.08
Adjusted^a,b^	0.51	0.15–0.88	<0.006	0.52	0.02–1.02	<0.05

^a^Adjusted for differences in age, sex, and education

^b^Analysis excluded data from two BD subjects with undetected *CRMP1* mRNA levels

## Discussion

The main finding of this study is the capture of dynamic molecular changes in response to lithium therapy, in association with symptomatic alterations, in the neurons of BD patients in vivo. Here we provide the first preliminary evidence implicating dysregulated neuronal expression of *CRMP1* in the neurobiology of BD. We have showed that decrements in *CRMP1* expression were significantly related to lithium-associated changes in the severity of depression and mania, suggesting that CRMP1 pathway might be involved in mood-stabilizing effects of lithium. A closely related protein, CRMP2, was recently reported to provide a possible mechanism for lithium’s neuroprotective effects, based on studies of neuronal cells derived from iPSCs of patients with BD^32^. Our study also extends previous reports that implicated dysregulated *GSK3β* in the pathophysiology of BD by providing experimental evidence that lithium may also affect its transcriptional regulation in neuronal tissues of patients with BD.

To address the molecular changes in neurons from patients with brain disorders, recent advances in stem cell biology, in particular the technology of iPSCs, have removed technical barriers to a reasonable extent. Nonetheless, the iPSCs technology can capture only “trait” changes, because, during the process of cell reprogramming and differentiation/conversion, the molecular signature that reflects the “state” at the time of biopsy is theoretically lost. By combining LCM and RT-PCR to study the transcriptional changes in the olfactory neurons of patients as they transition from one affective state to another following lithium monotherapy, our method provided us the rare opportunity to capture treatment-associated alterations in intraneuronal molecular markers that reflect these specific “state” changes.

The relatively small sample size for lithium-associated molecular changes is a limitation of this study that derives from our requirement that *OMP* expression in the LCM ‘captured neuroepithelial tissues’ was at least 2-fold greater than *OMP* expression in the undissected tissues from the same source. This requirement reduced the final sample size since biopsied specimens from some individuals did not meet that requirement. The sample size possibly explains the lack of statistical significance for *GSK3β* expression and its relation to changes in depressive symptoms. As shown in the power analysis in our Supplementary Figure 5, we are powered to determine molecular markers with strong effect sizes for mediational roles in the effect of lithium therapy on clinical response. Future studies with larger samples are needed to correctly identify molecular markers with moderate and weak effects.

One limitation regarding the ethical requirement to provide therapy for all symptomatic BD subjects is that state changes over the 6-week course of therapy could potentially occur spontaneously (i.e., irrespective of lithium). However, the mean duration of current episode in this sample (20.41 ± 20.02 weeks) is long enough to suggest that a good proportion of patients run a chronic course that may not resolve if lithium was not instituted. Moreover, as shown in Supplementary Figure 6, a 6-week treatment with lithium of six control subjects did not reproduce the same state-associated molecular changes in *CRMP1* or *GSK3β*, further supporting that the state-associated molecular changes in BD were not spurious effects of time. Even if clinical severity changes over time were resultant of spontaneous resolution, and not of lithium action, this study still reveals consistency in the direction of change between clinical severity and transcriptional levels over the 6-week period.

Additional limitations include the absence of a protein assay or the assessment of phosphorylation of GSK3β (which would be a direct reflection of GSK3β enzymatic activity) and the non-inclusion of other neuropsychiatric controls to determine the specificity of molecular markers. In the future, we plan to develop the olfactory experimental platform so that it may be available for protein assays^33–35^. Due to the limitation in the amount of RNA in the current state of the olfactory platform, we were forced to prioritize molecules for this study. While several isoforms of PKC are altered in BD, we chose only the isoform that is selectively inhibited by lithium and valproate, and one with known salience in transcriptional levels^8,36–38^. Similarly, the quantity of mRNA recovered from the olfactory neuronal tissue after LCM is not currently sufficient for genome-wide expression profiles. Nonetheless, it is becoming easier to obtain reliable transcriptomic data from small amounts of starting material with the maturation of next-generation sequencing technologies^39–41^. In future studies, we plan to study a broader group of isoforms and include other psychiatric populations (e.g., schizophrenia) in an unbiased manner.

In BD pathophysiology, the preponderance of evidence supporting GSK3β involvement comes from preclinical studies^42^, and from some molecular genetic studies in humans^43–45^. Studies examining the relationship between GSK3β and BD have yielded mixed results using peripheral blood cells^46–48^. Following recent evidence that lithium therapy reduces rat hippocampal mRNA levels of *GSK3β* both in vivo and in vitro^4^, our study answered a necessary question regarding whether lithium downregulates olfactory neuronal expression of *GSK3β* in patients with BD, and whether such a change is related to clinical outcome. Our discovery that baseline differences exist in the olfactory neuronal expression of *GSK3β* comparing BD subjects to controls, strengthened by the inverse correlation between post-treatment mania symptom severity and treatment-associated downregulation of *GSK3β* levels, supports a future extension to study in vitro treatment-associated gene and protein levels in olfactory neuronal cultures of patients with known clinical response to lithium, to determine which of these predicts clinical response more accurately. Although CRMPs have not been previously studied in clinical populations of BD, their role in neurodevelopment^49^, olfactory and hippocampal neuroplasticity^50^, and regulation of voltage-gated calcium channels^51,52^, as well as their functional designation as a molecular substrate of GSK3β protein, make them very attractive targets for investigation of mechanisms of BD and related neuropsychiatric disorders. Most CRMPs are exclusively expressed in neuronal tissues, particularly in sites with life-long neurogenesis, such as the olfactory epithelium and the OB, where CRMP1 is consistently expressed^25^. Prior to this study, we examined the expression of CRMP1 in lymphocytes, lymphoblast cell lines, and olfactory neuronal tissues of six BD subjects (used for estimating sample size for this study) and in controls. CRMP1 was expressed in the olfactory neurons of all six BD subjects, but not expressed in the lymphocytes of any of these patients or controls. We also found that CRMP1 was not detected in the lymphoblast cells of four out of six BD patients^26^. This supports the relevance of studying molecular markers of BD in neuronal platforms like ours.

Finally, we did not observe an association between BD and mRNA levels of *AKT1* or *PKCε*. A wealth of neurobiological literature, however, provides persuasive evidence for the relevance of activated (or inhibited) forms of these two molecules to BD and other major mental illnesses^5–7^. This evidence highlights the role of AKT1 and PKC*ε* in phosphorylation cascades that drive intracellular transduction signals relevant to BD. Lack of association between mRNA levels of these molecules and BD in this study may indicate that they may be post-translationally modified in their role in lithium therapy. As mentioned above, future studies will develop the olfactory experimental platform so that it may become available for protein assays and provide potential evidence for AKT1 and PKC post-translational modifications^34,35,53^.

## Electronic supplementary material

Supplementary Figure 1

Supplementary Figure 2

Supplementary Figure 3

Supplementary Figure 4

Supplementary Figure 5

Supplementary Figure 6A

Supplementary Figure 6B

Supplementary data

Supplementary Table1
