# Supplementary figures and images for: Lithium-associated transcriptional regulation of CRMP1 in patient-derived olfactory neurons and symptom changes in bipolar disorder
Source: Transl Psychiatry. 2018 Apr 18;8:81. doi: 10.1038/s41398-018-0126-6 (PMC5904136; doi:10.1038/s41398-018-0126-6)

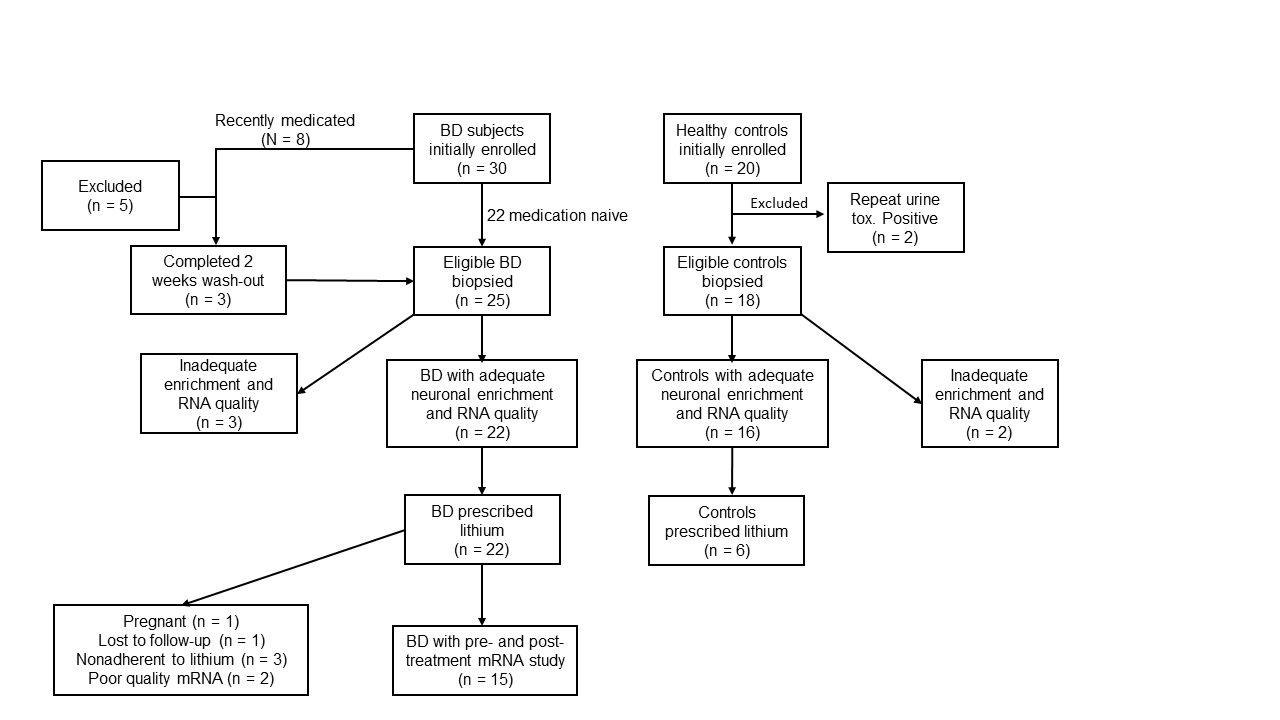

Supplement: Supplementary file 1 — Supplementary Figure 1 [file 41398_2018_126_MOESM1_ESM.tif]

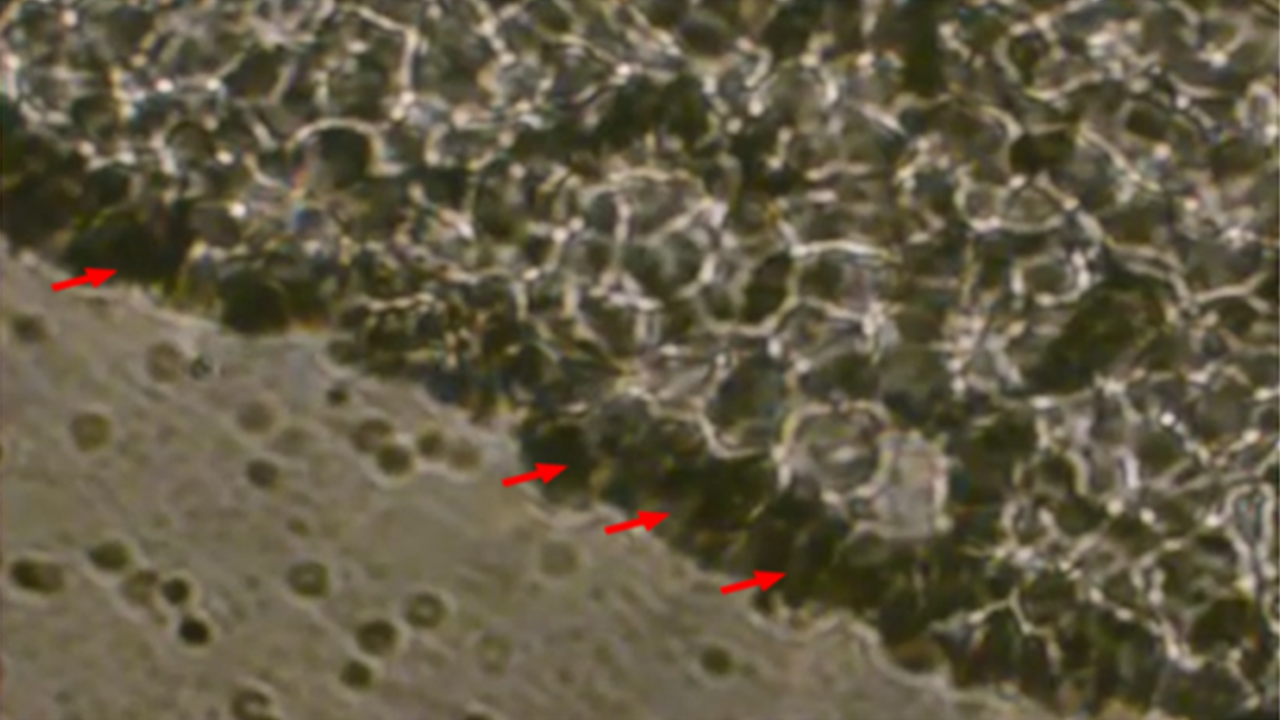

Supplement: Supplementary file 2 — Supplementary Figure 2 [file 41398_2018_126_MOESM2_ESM.tif]

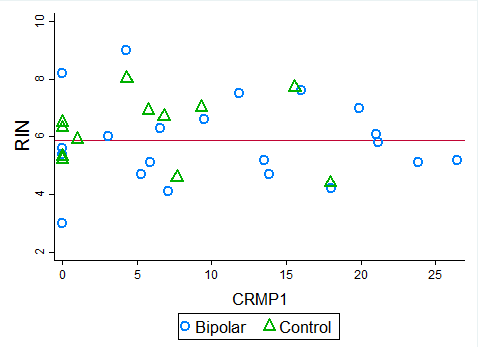

Supplement: Supplementary file 3 — Supplementary Figure 3 [file 41398_2018_126_MOESM3_ESM.tif]

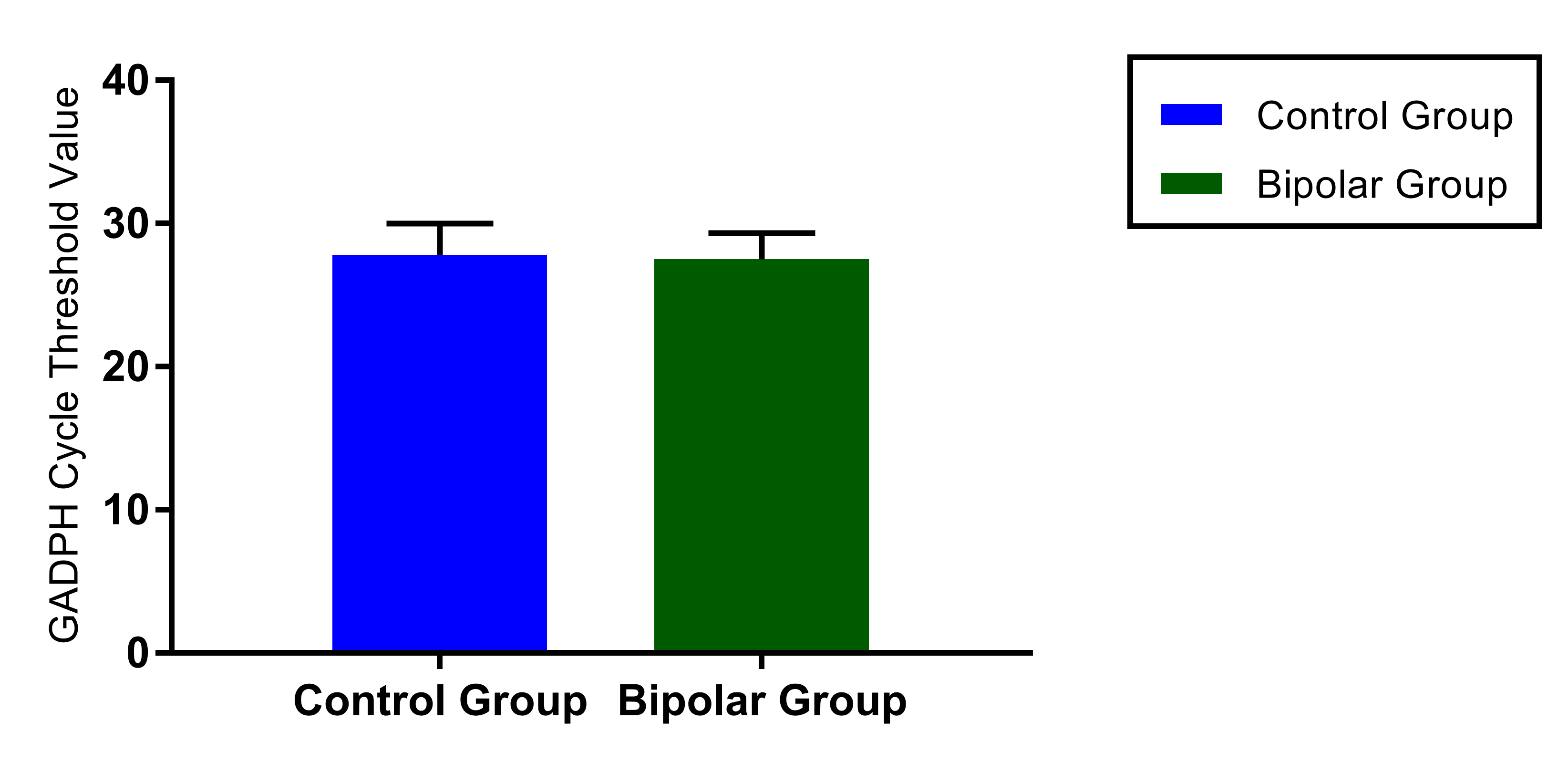

Supplement: Supplementary file 4 — Supplementary Figure 4 [file 41398_2018_126_MOESM4_ESM.tif]

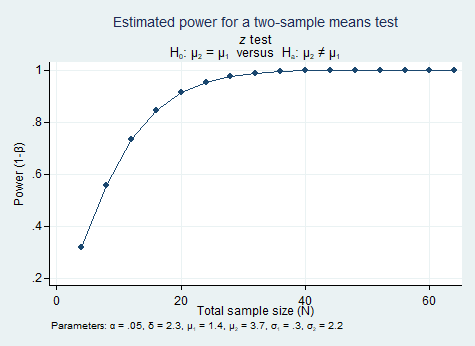

Supplement: Supplementary file 5 — Supplementary Figure 5 [file 41398_2018_126_MOESM5_ESM.tif]

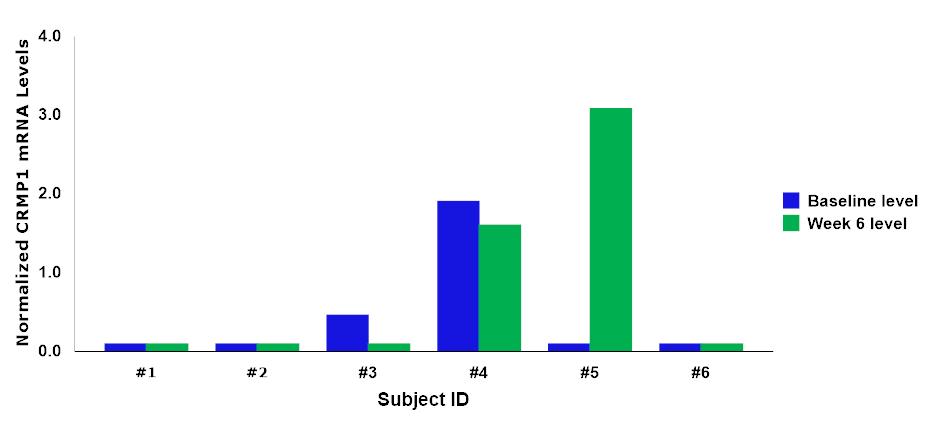

Supplement: Supplementary file 6 — Supplementary Figure 6A [file 41398_2018_126_MOESM6_ESM.tif]

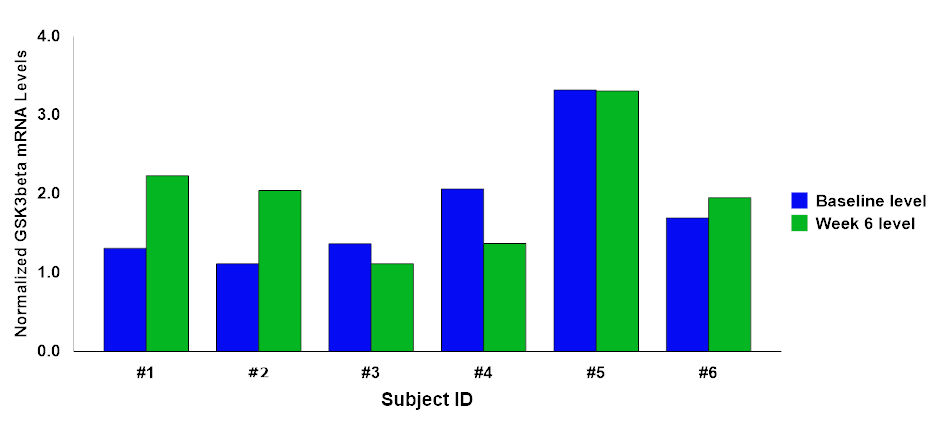

Supplement: Supplementary file 7 — Supplementary Figure 6B [file 41398_2018_126_MOESM7_ESM.tif]
