# Supplementary material for: Lithium-associated transcriptional regulation of CRMP1 in patient-derived olfactory neurons and symptom changes in bipolar disorder
Source: Transl Psychiatry. 2018 Apr 18;8:81. doi: 10.1038/s41398-018-0126-6 (PMC5904136; doi:10.1038/s41398-018-0126-6)
Supplement: Supplementary file 8 — Supplementary data [file 41398_2018_126_MOESM8_ESM.docx]

**SUPPLEMENTARY DATA**

**FIGURES**

**
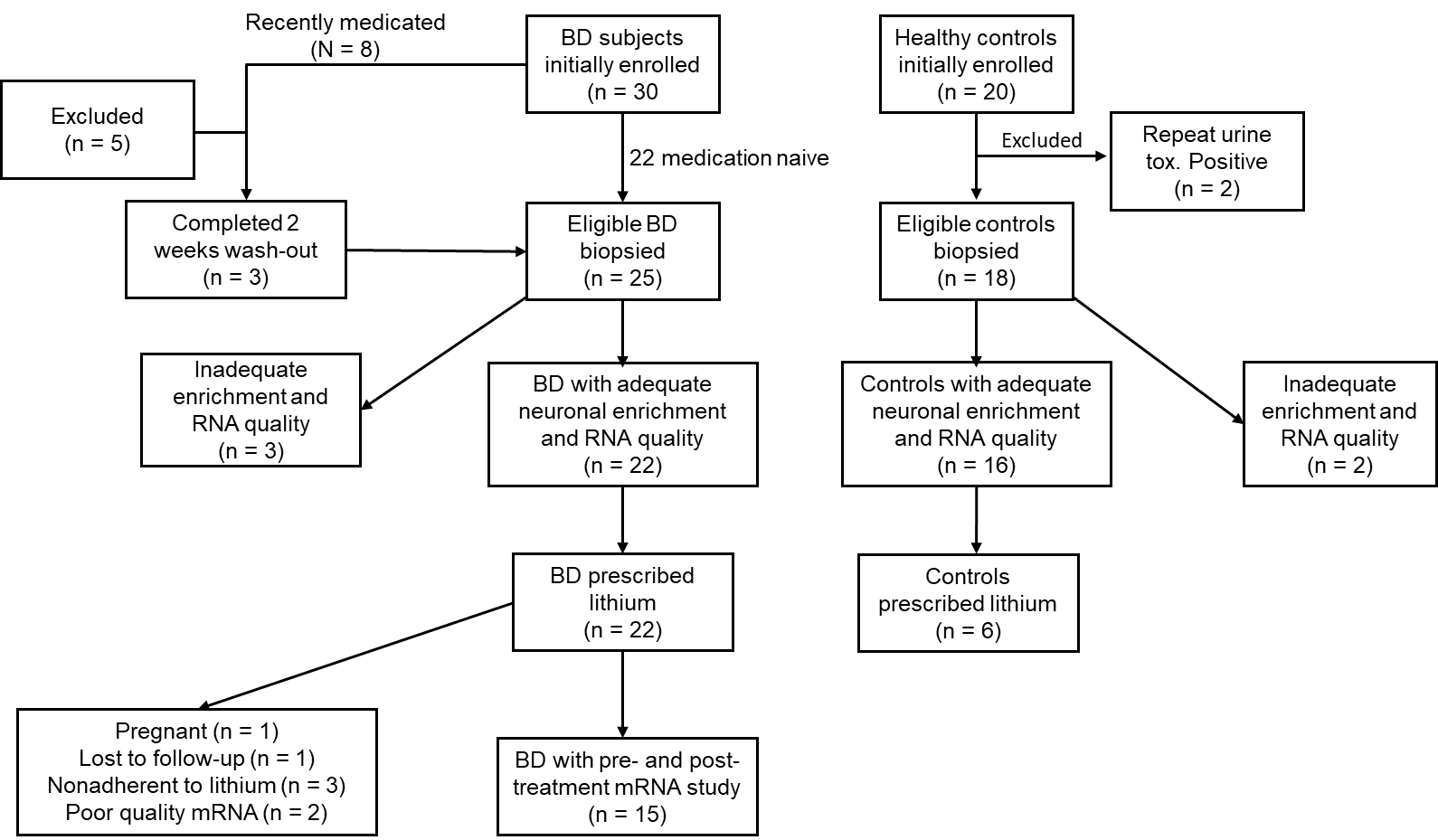
SUPPLEMENTARY FIGURE 1 Study Flowchart**

**Legend for Supplementary Figure 1:** Out of 30 BD subjects screened 22 were treatment naïve, and 3 of the previously medicated BD subjects joined the study after a two-week washout. Three of the 25 BD subjects had poor RNA or low expression of OMP, and were excluded from baseline studies. Sixteen out of 18 eligible controls had quality specimens for baseline study. All 22 BD subjects were prescribed lithium, but 5 were excluded for poor adherence, pregnancy or loss to follow-up; and 2 had low quality specimen. Therefore, 15 out of the eligible 22 at baseline had RNA and clinical data post-treatment for analysis.

**Supplementary Figure 2: Olfactory Neuroepithelium from Photoactivated Localization Microscopy (PALM) Microscope**


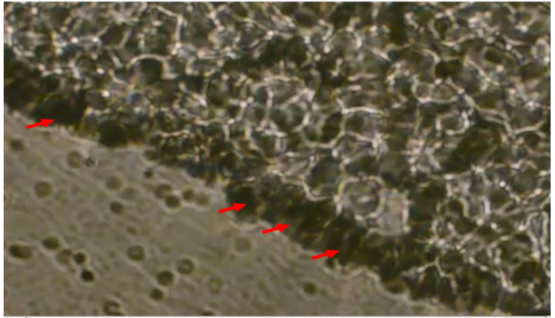


**Legend for Supplementary Figure 2:** Olfactory epithelial layer depicted by Red arrows.

**Supplementary Figure 3. Correlation between MRNA levels of CRMP1 and RIN values in BD cases and controls**


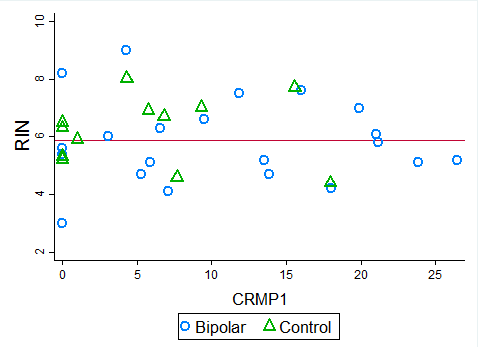


**Legend for Supplementary Figure 3:** Correlation between RIN and CRMP1 levels is ~0 (r = 0.002, p< 1.0). Data shown separately for BD subjects (Blue circle) and healthy controls (Green triangle) indicates no disease group-specific correlation with RIN

**Supplementary Figure 4: Expression Levels of GADPH Gene in Olfactory Epithelium of Bipolar Patients and Controls**

**

**

**Legend for Supplementary Figure 4:** GADPH ct values in BD patients and controls. Mean (± S.D.) *GADPH* ct values for BD vs. controls were 27.52 (1.83) and 27.83 (2.19), respectively (P<0.6).

**
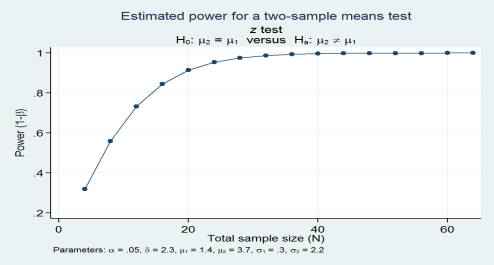
Supplementary Figure 5: Power and Sample Size**

**Legend for Supplementary Figure 5:** Power analysis for difference in *GSK3β* levels between cases and controls generated pre-study. A total sample size of 20 provides more than 80% to detect group differences in *GSK3β* levels.

**Supplementary Figure 6: mRNA Levels in Control Subjects**

**
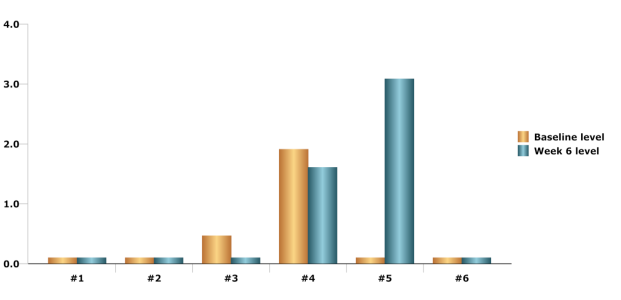
*6A Baseline and 6th week normalized mRNA levels for CRMP1***


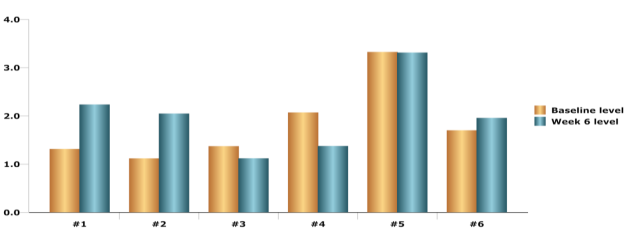
***6B Baseline and 6th week normalized mRNA levels for GSK3β***

**Legend for Supplementary Figure 6:** Baseline and 6th week normalized mRNA levels for *GSK3β* and *CRMP1* in 6 control subjects. These did not reveal a pattern of reduction of the second (i.e. 6th week) mRNA levels from their baseline levels, as seen in subjects with BD treated with Lithium.

**Supplementary Table 1**. Linear associations between pre- and post-treatment lithium levels and within-individual (pre- and post-treatment) changes in mRNA levels of *GSK3β* and *CRMP1*

|  | **GSK3β** | | | **CRMP1** | | |
| --- | --- | --- | --- | --- | --- | --- |
|  | β | 95% CI | p | β | 95% CI | p |
| Treatment | -1.11 | -1.35 – -0.40 | <0.001 | -8.41 | -13.11 – -3.03 | <0.002 |
| Lithium levels | -0.69 | -1.28 – -0.11 | <0.02 | -5.89 | -11.61 – -0.18 | <0.05 |
