# Supplementary material for: Lithium-associated transcriptional regulation of CRMP1 in patient-derived olfactory neurons and symptom changes in bipolar disorder
Source: Transl Psychiatry. 2018 Apr 18;8:81. doi: 10.1038/s41398-018-0126-6 (PMC5904136; doi:10.1038/s41398-018-0126-6)
Supplement: Supplementary file 9 — Supplementary Table1 [file 41398_2018_126_MOESM9_ESM.docx]

**Supplementary Table 1**. Linear associations between pre- and post-treatment lithium levels and within-individual (pre- and post-treatment) changes in mRNA levels of *GSK3β* and *CRMP1*

|  | **GSK3β** | | | **CRMP1** | | |
| --- | --- | --- | --- | --- | --- | --- |
|  | β | 95% CI | p | β | 95% CI | p |
| Treatment | -1.11 | -1.35 – -0.40 | <0.001 | -8.41 | -13.11 – -3.03 | <0.002 |
| Lithium levels | -0.69 | -1.28 – -0.11 | <0.02 | -5.89 | -11.61 – -0.18 | <0.05 |
